# Supplementary material for: An International Comparison of Presentation, Outcomes and CORONET Predictive Score Performance in Patients with Cancer Presenting with COVID-19 across Different Pandemic Waves
Source: Cancers (Basel). 2022 Aug 16;14(16):3931. doi: 10.3390/cancers14163931 (PMC9406013; doi:10.3390/cancers14163931)
Supplement: Supplementary file 1 [file cancers-14-03931-s001.zip › cancers-1790646-supplementary.pdf]

Supplementary Materials

Supplementary Table S1. Number of patients in the dataset obtained from 12 participating hospitals in the UK, 2 hospitals in Spain, 4 hospitals in the USA, and as part of the ESMO-CoCARE registry, hospitals throughout the world, excluding the USA, Canada and Latin America.

Cohort	Total	1 D614G	2 Alpha	4 Omicron
**United Kingdom**	583	416	131	36
**Spain**	239	119	93	27
**ESMO Co-Care**	667	416	251	
**USA**	194	194		
**France**	178	178		
**Denmark**	107	107		
**Total**	1968	1430	475	63

Supplementary Table S2. Known features associated with COVID-19 severity compared between different waves, stratified by outcome.

Feature	Wave	Outcomes	*p*-Value	Significance
Age	1	Discharged vs Admitted	0.0102	*
		Discharged vs Admitted+O2	0.0000	***
		Discharged vs Admitted+O2+died	0.0000	***
		Admitted vs Admitted+O2	0.0000	***
		Admitted vs Admitted+O2+died	0.0000	***
		Admitted+O2 vs Admitted+O2+died	0.0055	**
	2	Discharged vs Admitted+O2	0.0000	***
		Discharged vs Admitted+O2+died	0.0000	***
		Admitted vs Admitted+O2	0.0247	*
		Admitted vs Admitted+O2+died	0.0037	**
Solid cancer stage	1	Discharged vs Admitted	0.0000	***
		Discharged vs Admitted+O2+died	0.0000	***
		Admitted vs Admitted+O2	0.0099	**
		Admitted vs Admitted+O2+died	0.0000	***
		Admitted+O2 vs Admitted+O2+died	0.0001	***
	2	Discharged vs Admitted	0.0215	*
		Discharged vs Admitted+O2	0.0001	***
		Discharged vs Admitted+O2+died	0.0002	***
NEWS2	1	Discharged vs Admitted	0.0045	**
		Discharged vs Admitted+O2	0.0000	***
		Discharged vs Admitted+O2+died	0.0000	***
		Admitted vs Admitted+O2	0.0000	***
		Admitted vs Admitted+O2+died	0.0000	***
	2	Discharged vs Admitted	0.0214	*
		Discharged vs Admitted+O2	0.0026	**
		Discharged vs Admitted+O2+died	0.0000	***
		Admitted vs Admitted+O2+died	0.0000	***
		Admitted+O2 vs Admitted+O2+died	0.0000	***
	4	Discharged vs Admitted+O2	0.0013	**
		Admitted vs Admitted+O2	0.0049	**
Performance status	1	Discharged vs Admitted	0.0009	***
		Discharged vs Admitted+O2	0.0000	***
		Discharged vs Admitted+O2+died	0.0000	***
		Admitted vs Admitted+O2+died	0.0000	***
		Admitted+O2 vs Admitted+O2+died	0.0002	***
Total no. comorbidities	1	Discharged vs Admitted+O2	0.0000	***
		Discharged vs Admitted+O2+died	0.0000	***
		Admitted vs Admitted+O2	0.0008	***
		Admitted vs Admitted+O2+died	0.0000	***
	2	Discharged vs Admitted+O2+died	0.0000	***
		Admitted vs Admitted+O2+died	0.0001	***
		Admitted+O2 vs Admitted+O2+died	0.0018	**
Albumin	1	Discharged vs Admitted	0.0005	***
		Discharged vs Admitted+O2	0.0000	***
		Discharged vs Admitted+O2+died	0.0000	***
		Admitted vs Admitted+O2	0.0000	***
		Admitted vs Admitted+O2+died	0.0000	***
	2	Discharged vs Admitted	0.0134	*
		Discharged vs Admitted+O2	0.0000	***
		Discharged vs Admitted+O2+died	0.0000	***
		Admitted vs Admitted+O2	0.0000	***
		Admitted vs Admitted+O2+died	0.0000	***
	4	Discharged vs Admitted+O2	0.0060	**

Platelets	1	Discharged vs Admitted+O2	0.0449	*
		Discharged vs Admitted+O2+died	0.0012	**
		Admitted vs Admitted+O2+died	0.0012	**
	2	Discharged vs Admitted+O2+died	0.0132	*
CRP	1	Discharged vs Admitted	0.0000	***
		Discharged vs Admitted+O2	0.0000	***
		Discharged vs Admitted+O2+died	0.0000	***
		Admitted vs Admitted+O2	0.0000	***
		Admitted vs Admitted+O2+died	0.0000	***
		Admitted+O2 vs Admitted+O2+died	0.0089	**
	2	Discharged vs Admitted	0.0139	*
		Discharged vs Admitted+O2	0.0000	***
		Discharged vs Admitted+O2+died	0.0000	***
		Admitted vs Admitted+O2	0.0012	**
		Admitted vs Admitted+O2+died	0.0000	***
		Admitted+O2 vs Admitted+O2+died	0.0118	*
	4	Discharged vs Admitted	0.0117	*
		Discharged vs Admitted+O2	0.0002	***
Lymphocyte	1	Discharged vs Admitted	0.0027	**
		Discharged vs Admitted+O2	0.0062	**
		Discharged vs Admitted+O2+died	0.0000	***
		Admitted vs Admitted+O2+died	0.0036	**
		Admitted+O2 vs Admitted+O2+died	0.0018	**
	2	Discharged vs Admitted	0.0035	**
		Discharged vs Admitted+O2	0.0000	***
		Discharged vs Admitted+O2+died	0.0000	***
		Admitted vs Admitted+O2+died	0.0428	*
Neutrophil	1	Discharged vs Admitted+O2	0.0000	***
		Discharged vs Admitted+O2+died	0.0000	***
		Admitted vs Admitted+O2	0.0112	*
		Admitted vs Admitted+O2+died	0.0001	***
	2	Discharged vs Admitted+O2+died	0.0037	**
	4	Discharged vs Admitted+O2	0.0176	*
NLR	1	Discharged vs Admitted	0.0171	*
		Discharged vs Admitted+O2	0.0000	***
		Discharged vs Admitted+O2+died	0.0000	***
		Admitted vs Admitted+O2+died	0.0000	***
		Admitted+O2 vs Admitted+O2+died	0.0014	**
	2	Discharged vs Admitted	0.0010	**
		Discharged vs Admitted+O2	0.0002	***
		Discharged vs Admitted+O2+died	0.0000	***
		Admitted vs Admitted+O2+died	0.0039	**
		Admitted+O2 vs Admitted+O2+died	0.0133	*

Statistically significant differences between features within waves were marked by: *** - p <0.001, ** - p <0.01, * - p <0.05; Mann-Whitney U and Chi^2 tests were used for numeric and categorical (solid cancer stage and performance status) features accordingly. p values were adjusted using Benjamini–Hochberg.

Supplementary Table S3. Number of patients selected for CORONET validation before and after exclusion based on the criteria for missing values.

	No. patients in cohorts			No. patients used to evaluate CORONET		
**Country**	**1 D614G**	**2 Alpha**	**4 Omicron**	**1 D614G**	**2 Alpha**	**4 Omicron**
France	178			123 (69.1%)		
Denmark	107			92 (86%)		
UK	43	31	36	43 (100%)	30 (96.8%)	27 (75%)
Spain		20	27		18 (90%)	27 (100%)
Total	328	51	63	258 (78.7%)	48 (94.1%)	54 (85.7%)

Supplementary Table S4. Predicted CORONET score for patients from wave D614G, Alpha and Omicron waves.

	CORONET score, median [Q1,Q3]			
**Outcome**	**1 D614G**	**2 Alpha**	**4 Omicron**	**Significant differences between waves***
**Discharged**	0.8 [0.48,1.34]	1.01 [0.64,1.16]	0.68 [0.57,0.93]	ns
**Admitted**	1.35 [0.88,1.81]	1.11 [0.86,1.29]	1.21 [0.67,1.32]	ns
**Admitted+O$_2$**	1.92 [1.24,2.18]	1.5 [1.09,2.12]	1.89 [1.41,2.13]	ns
**Admitted+O$_2$+died**	2.01 [1.57,2.38]	1.67 [1.38,1.74]	-	ns

* ns – non-significant, p-val > 0.05, Mann-Whitney U, p-values corrected for multiple testing using one-step Sidak correction.

Supplementary Table S5. Treatments for COVID-19 amongst patients used to evaluate CORONET.

	1 D614G			2 Alpha			4 Omicron		
	**No. Patients treated**	**No. Patients not treated**	**missing**	**No. Patients treated**	**No. Patients not treated**	**missing**	**No. Patients treated**	**No. Patients not treated**	**missing**
steroids	3 (21.4%)	11 (78.6%)	314	26 (54.2%)	22 (45.8%)	3	19 (41.3%)	27 (58.7%)	0
remdesivir	1 (7.1%)	13 (92.9%)	314	3 (6.4%)	44 (93.6%)	4	0 (0%)	45 (100%)	1
lipinovir	0 (0%)	14 (100%)	314	0 (0%)	47 (100%)	4	0 (0%)	46 (100%)	0
interferon	0 (0%)	14 (100%)	314	0 (0%)	47 (100%)	4	0 (0%)	46 (100%)	0
interferon beta	1 (7.1%)	13 (92.9%)	314	0 (0%)	47 (100%)	4	0 (0%)	46 (100%)	0
anticoagulation prophyl	12 (85.7%)	2 (14.3%)	314	24 (50%)	24 (50%)	3	22 (47.8%)	24 (52.2%)	0
anticoagulation treat	0 (0%)	14 (100%)	314	3 (6.5%)	43 (93.5%)	5	0 (0%)	46 (100%)	0
antibiotic	10 (71.4%)	4 (28.6%)	314	30 (62.5%)	18 (37.5%)	3	17 (37%)	29 (63%)	0
plasma	0 (0%)	14 (100%)	314	0 (0%)	47 (100%)	4	0 (0%)	46 (100%)	0
tocilizumab	0 (0%)	14 (100%)	314	4 (8.5%)	43 (91.5%)	4	2 (4.3%)	44 (95.7%)	0
nebulised interferonb	0 (0%)	14 (100%)	314	0 (0%)	47 (100%)	4	0 (0%)	46 (100%)	0
hydroxychloroquine	0 (0%)	14 (100%)	314	0 (0%)	47 (100%)	4	0 (0%)	46 (100%)	0
aspirin	0 (0%)	14 (100%)	314	1 (2.1%)	46 (97.9%)	4	0 (0%)	46 (100%)	0
baricitinib	0 (0%)	14 (100%)	314	0 (0%)	47 (100%)	4	0 (0%)	46 (100%)	0
molnupiravir			328			51	0 (0%)	42 (100%)	4
sotrovimab			328			51	0 (0%)	42 (100%)	4
other drug			328			51			46

Supplementary Table S6. Sensitivity, specificity, Positive Predictive Value and Negative Predictive Value for admission, requirement for oxygen in relation to the threshold defined by the CORONET score for 258 patients from D614G wave. Row highlighted in grey signifies the admission threshold.

		Admission									Requirement for O2								
**threshold**	**Total # of pts above threshold**	**TN**	**FN**	**TP**	**FP**	**Sensitivity**	**Specificity**	**PPV**	**NPV**	**Accuracy**	**TN**	**FN**	**TP**	**FP**	**Sensitivity**	**Specificity**	**PPV**	**NPV**	**Accuracy**
>= 0.2	258	0	0	216	42	1.00	0.00	0.84	-	0.84	0	0	110	148	1.00	0.00	0.43	-	0.43
>= 0.3	256	1	1	215	41	1.00	0.02	0.84	0.50	0.84	2	0	110	146	1.00	0.01	0.43	1.00	0.43
>= 0.4	245	8	5	211	34	0.98	0.19	0.86	0.62	0.85	13	0	110	135	1.00	0.09	0.45	1.00	0.48
>= 0.5	238	12	8	208	30	0.96	0.29	0.87	0.60	0.85	20	0	110	128	1.00	0.14	0.46	1.00	0.50
>= 0.6	227	16	15	201	26	0.93	0.38	0.89	0.52	0.84	30	1	109	118	0.99	0.20	0.48	0.97	0.54
>= 0.7	220	18	20	196	24	0.91	0.43	0.89	0.47	0.83	37	1	109	111	0.99	0.25	0.50	0.97	0.57
>= 0.8	210	22	26	190	20	0.88	0.52	0.90	0.46	0.82	46	2	108	102	0.98	0.31	0.51	0.96	0.60
>= 0.9	200	27	31	185	15	0.86	0.64	0.93	0.47	0.82	53	5	105	95	0.95	0.36	0.53	0.91	0.61
>= 1.0	189	28	41	175	14	0.81	0.67	0.93	0.41	0.79	62	7	103	86	**0.94**	0.42	0.55	0.90	0.64
>= 1.1	182	29	47	169	13	0.78	0.69	0.93	0.38	0.77	67	9	101	81	0.92	0.45	0.55	0.88	0.65
>= 1.2	174	31	53	163	11	0.75	0.74	0.94	0.37	0.75	72	12	98	76	0.89	0.49	0.56	0.86	0.66
>= 1.3	167	32	59	157	10	0.73	0.76	0.94	0.35	0.73	76	15	95	72	0.86	0.51	0.57	0.84	0.66
>= 1.4	159	32	67	149	10	0.69	0.76	0.94	0.32	0.70	83	16	94	65	0.85	0.56	0.59	0.84	0.69
>= 1.5	151	33	74	142	9	0.66	0.79	0.94	0.31	0.68	86	21	89	62	0.81	0.58	0.59	0.80	0.68
>= 1.6	136	35	87	129	7	0.60	0.83	0.95	0.29	0.64	96	26	84	52	0.76	0.65	0.62	0.79	0.70
>= 1.7	127	36	95	121	6	0.56	0.86	0.95	0.27	0.61	102	29	81	46	0.74	0.69	0.64	0.78	0.71
>= 1.8	115	37	106	110	5	0.51	0.88	0.96	0.26	0.57	109	34	76	39	0.69	0.74	0.66	0.76	0.72
>= 1.9	101	40	117	99	2	0.46	0.95	0.98	0.25	0.54	118	39	71	30	0.65	0.80	0.70	0.75	0.73
>= 2.0	82	41	135	81	1	0.38	0.98	0.99	0.23	0.47	124	52	58	24	0.53	0.84	0.71	0.70	0.71
>= 2.1	64	41	153	63	1	0.29	0.98	0.98	0.21	0.40	126	68	42	22	0.38	0.85	0.66	0.65	0.65
>= 2.2	52	41	165	51	1	0.24	0.98	0.98	0.20	0.36	130	76	34	18	0.31	0.88	0.65	0.63	0.64
>= 2.3	45	41	172	44	1	0.20	0.98	0.98	0.19	0.33	133	80	30	15	0.27	0.90	0.67	0.62	0.63
>= 2.4	35	41	182	34	1	0.16	0.98	0.97	0.18	0.29	135	88	22	13	0.20	0.91	0.63	0.61	0.61
>= 2.5	24	42	192	24	0	0.11	1.00	1.00	0.18	0.26	137	97	13	11	0.12	0.93	0.54	0.59	0.58
>= 2.6	16	42	200	16	0	0.07	1.00	1.00	0.17	0.22	142	100	10	6	0.09	0.96	0.63	0.59	0.59
>= 2.7	6	42	210	6	0	0.03	1.00	1.00	0.17	0.19	147	105	5	1	0.05	0.99	0.83	0.58	0.59
>= 2.8	2	42	214	2	0	0.01	1.00	1.00	0.16	0.17	147	109	1	1	0.01	0.99	0.50	0.57	0.57
>= 2.9	0	42	216	0	0	0.00	1.00	-	0.16	0.16	148	110	0	0	0.00	1.00	-	0.57	0.57

Supplementary Table S7. Sensitivity, specificity, Positive Predictive Value and Negative Predictive Value for admission, requirement for oxygen in relation to the threshold defined by the CORONET score for 48 patients from Alpha wave. Row highlighted in grey signifies the admission threshold.

		Admission									Requirement for O2								
**threshold**	**Total # of pts above threshold**	**TN**	**FN**	**TP**	**FP**	**Sensitivity**	**Specificity**	**PPV**	**NPV**	**Accuracy**	**TN**	**FN**	**TP**	**FP**	**Sensitivity**	**Specificity**	**PPV**	**NPV**	**Accuracy**
>= 0.2	48	0	0	40	8	1.00	0.00	0.83	-	0.83	0	0	27	21	1.00	0.00	0.56	-	0.56
>= 0.3	48	0	0	40	8	1.00	0.00	0.83	-	0.83	0	0	27	21	1.00	0.00	0.56	-	0.56
>= 0.4	48	0	0	40	8	1.00	0.00	0.83	-	0.83	0	0	27	21	1.00	0.00	0.56	-	0.56
>= 0.5	46	1	1	39	7	0.98	0.13	0.85	0.50	0.83	2	0	27	19	1.00	0.10	0.59	1.00	0.60
>= 0.6	44	2	2	38	6	0.95	0.25	0.86	0.50	0.83	4	0	27	17	1.00	0.19	0.61	1.00	0.65
>= 0.7	42	3	3	37	5	0.93	0.38	0.88	0.50	0.83	6	0	27	15	1.00	0.29	0.64	1.00	0.69
>= 0.8	41	3	4	36	5	0.90	0.38	0.88	0.43	0.81	6	1	26	15	0.96	0.29	0.63	0.86	0.67
>= 0.9	37	3	8	32	5	0.80	0.38	0.86	0.27	0.73	8	3	24	13	0.89	0.38	0.65	0.73	0.67
>= 1.0	35	4	9	31	4	0.78	0.50	0.89	0.31	0.73	10	3	24	11	**0.89**	0.48	0.69	0.77	0.71
>= 1.1	31	5	12	28	3	0.70	0.63	0.90	0.29	0.69	11	6	21	10	0.78	0.52	0.68	0.65	0.67
>= 1.2	27	6	15	25	2	0.63	0.75	0.93	0.29	0.65	14	7	20	7	0.74	0.67	0.74	0.67	0.71
>= 1.3	22	7	19	21	1	0.53	0.88	0.95	0.27	0.58	17	9	18	4	0.67	0.81	0.82	0.65	0.73
>= 1.4	19	7	22	18	1	0.45	0.88	0.95	0.24	0.52	18	11	16	3	0.59	0.86	0.84	0.62	0.71
>= 1.5	17	7	24	16	1	0.40	0.88	0.94	0.23	0.48	18	13	14	3	0.52	0.86	0.82	0.58	0.67
>= 1.6	14	7	27	13	1	0.33	0.88	0.93	0.21	0.42	19	15	12	2	0.44	0.90	0.86	0.56	0.65
>= 1.7	13	7	28	12	1	0.30	0.88	0.92	0.20	0.40	19	16	11	2	0.41	0.90	0.85	0.54	0.63
>= 1.8	11	7	30	10	1	0.25	0.88	0.91	0.19	0.35	19	18	9	2	0.33	0.90	0.82	0.51	0.58
>= 1.9	11	7	30	10	1	0.25	0.88	0.91	0.19	0.35	19	18	9	2	0.33	0.90	0.82	0.51	0.58
>= 2.0	11	7	30	10	1	0.25	0.88	0.91	0.19	0.35	19	18	9	2	0.33	0.90	0.82	0.51	0.58
>= 2.1	8	7	33	7	1	0.18	0.88	0.88	0.18	0.29	20	20	7	1	0.26	0.95	0.88	0.50	0.56
>= 2.2	4	8	36	4	0	0.10	1.00	1.00	0.18	0.25	21	23	4	0	0.15	1.00	1.00	0.48	0.52
>= 2.3	4	8	36	4	0	0.10	1.00	1.00	0.18	0.25	21	23	4	0	0.15	1.00	1.00	0.48	0.52
>= 2.4	3	8	37	3	0	0.08	1.00	1.00	0.18	0.23	21	24	3	0	0.11	1.00	1.00	0.47	0.50
>= 2.5	2	8	38	2	0	0.05	1.00	1.00	0.17	0.21	21	25	2	0	0.07	1.00	1.00	0.46	0.48
>= 2.6	2	8	38	2	0	0.05	1.00	1.00	0.17	0.21	21	25	2	0	0.07	1.00	1.00	0.46	0.48
>= 2.7	2	8	38	2	0	0.05	1.00	1.00	0.17	0.21	21	25	2	0	0.07	1.00	1.00	0.46	0.48
>= 2.8	0	8	40	0	0	0.00	1.00	-	0.17	0.17	21	27	0	0	0.00	1.00	-	0.44	0.44
>= 2.9	0	8	40	0	0	0.00	1.00	-	0.17	0.17	21	27	0	0	0.00	1.00	-	0.44	0.44

Supplementary Table S8. Sensitivity, specificity, Positive Predictive Value and Negative Predictive Value for admission, requirement for oxygen in relation to the threshold defined by the CORONET score for 54 patients from Omicron wave. Row highlighted in grey signifies the admission threshold.

threshold	Total # of pts above thresh-old	Admission									Requirement for O2								
		TN	FN	TP	FP	Sensitivity	Specificity	PPV	NPV	Accuracy	TN	FN	TP	FP	Sensitivity	Specificity	PPV	NPV	Accuracy
>= 0.2	54	0	0	36	18	1.00	0.00	0.67	-	0.67	0	0	18	36	1.00	0.00	0.33	-	0.33
>= 0.3	54	0	0	36	18	1.00	0.00	0.67	-	0.67	0	0	18	36	1.00	0.00	0.33	-	0.33
>= 0.4	52	1	1	35	17	0.97	0.06	0.67	0.50	0.67	2	0	18	34	1.00	0.06	0.35	1.00	0.37
>= 0.5	49	2	3	33	16	0.92	0.11	0.67	0.40	0.65	5	0	18	31	1.00	0.14	0.37	1.00	0.43
>= 0.6	45	6	3	33	12	0.92	0.33	0.73	0.67	0.72	9	0	18	27	1.00	0.25	0.40	1.00	0.50
>= 0.7	40	9	5	31	9	0.86	0.50	0.78	0.64	0.74	14	0	18	22	1.00	0.39	0.45	1.00	0.59
>= 0.8	35	11	8	28	7	0.78	0.61	0.80	0.58	0.72	18	1	17	18	0.94	0.50	0.49	0.95	0.65
>= 0.9	32	12	10	26	6	0.72	0.67	0.81	0.55	0.70	20	2	16	16	0.89	0.56	0.50	0.91	0.67
>= 1.0	29	15	10	26	3	0.72	0.83	0.90	0.60	0.76	23	2	16	13	0.89	0.64	0.55	0.92	0.72
>= 1.1	28	15	11	25	3	0.69	0.83	0.89	0.58	0.74	23	3	15	13	0.83	0.64	0.54	0.88	0.70
>= 1.2	26	16	12	24	2	0.67	0.89	0.92	0.57	0.74	25	3	15	11	0.83	0.69	0.58	0.89	0.74
>= 1.3	23	16	15	21	2	0.58	0.89	0.91	0.52	0.69	28	3	15	8	0.83	0.78	0.65	0.90	0.80
>= 1.4	19	16	19	17	2	0.47	0.89	0.89	0.46	0.61	30	5	13	6	0.72	0.83	0.68	0.86	0.80
>= 1.5	16	17	21	15	1	0.42	0.94	0.94	0.45	0.59	32	6	12	4	0.67	0.89	0.75	0.84	0.81
>= 1.6	14	18	22	14	0	0.39	1.00	1.00	0.45	0.59	34	6	12	2	0.67	0.94	0.86	0.85	0.85
>= 1.7	13	18	23	13	0	0.36	1.00	1.00	0.44	0.57	34	7	11	2	0.61	0.94	0.85	0.83	0.83
>= 1.8	12	18	24	12	0	0.33	1.00	1.00	0.43	0.56	34	8	10	2	0.56	0.94	0.83	0.81	0.81
>= 1.9	10	18	26	10	0	0.28	1.00	1.00	0.41	0.52	34	10	8	2	0.44	0.94	0.80	0.77	0.78
>= 2.0	7	18	29	7	0	0.19	1.00	1.00	0.38	0.46	35	12	6	1	0.33	0.97	0.86	0.74	0.76
>= 2.1	6	18	30	6	0	0.17	1.00	1.00	0.38	0.44	35	13	5	1	0.28	0.97	0.83	0.73	0.74
>= 2.2	4	18	32	4	0	0.11	1.00	1.00	0.36	0.41	35	15	3	1	0.17	0.97	0.75	0.70	0.70
>= 2.3	2	18	34	2	0	0.06	1.00	1.00	0.35	0.37	36	16	2	0	0.11	1.00	1.00	0.69	0.70
>= 2.4	1	18	35	1	0	0.03	1.00	1.00	0.34	0.35	36	17	1	0	0.06	1.00	1.00	0.68	0.69
>= 2.5	1	18	35	1	0	0.03	1.00	1.00	0.34	0.35	36	17	1	0	0.06	1.00	1.00	0.68	0.69
>= 2.6	0	18	36	0	0	0.00	1.00	-	0.33	0.33	36	18	0	0	0.00	1.00	-	0.67	0.67
>= 2.7	0	18	36	0	0	0.00	1.00	-	0.33	0.33	36	18	0	0	0.00	1.00	-	0.67	0.67
>= 2.8	0	18	36	0	0	0.00	1.00	-	0.33	0.33	36	18	0	0	0.00	1.00	-	0.67	0.67
>= 2.9	0	18	36	0	0	0.00	1.00	-	0.33	0.33	36	18	0	0	0.00	1.00	-	0.67	0.67
